# Supplementary figures and images for: The physiological landscape and specificity of antibody repertoires are consolidated by multiple immunizations
Source: eLife. 2024 Dec 18;13:e92718. doi: 10.7554/eLife.92718 (PMC11655063; doi:10.7554/eLife.92718)

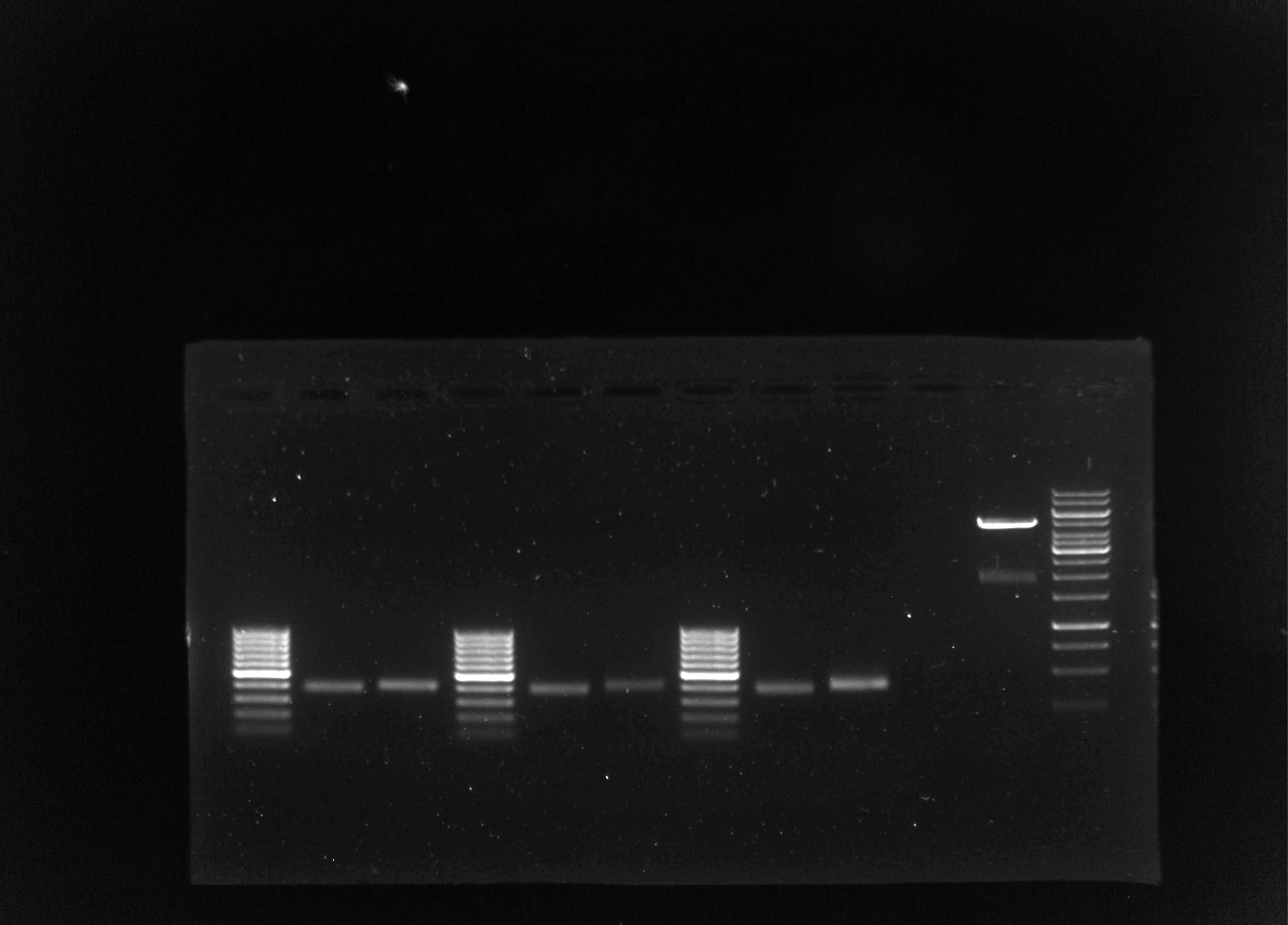

Supplement: Figure 5—figure supplement 1—source data 1. — Agarose gel electrophoresis (1%) of PCR-amplified VL and IgG VH genes from bone marrow (BM) samples of cohort-3x mice (3x-D, 3x-E, 3x-F), with 100 bp DNA size marker. [file elife-92718-fig5-figsupp1-data1.zip › Figure 5-figure supplement 1-source data 1/Figure 5-figure supplement 1-source data 1.tif]

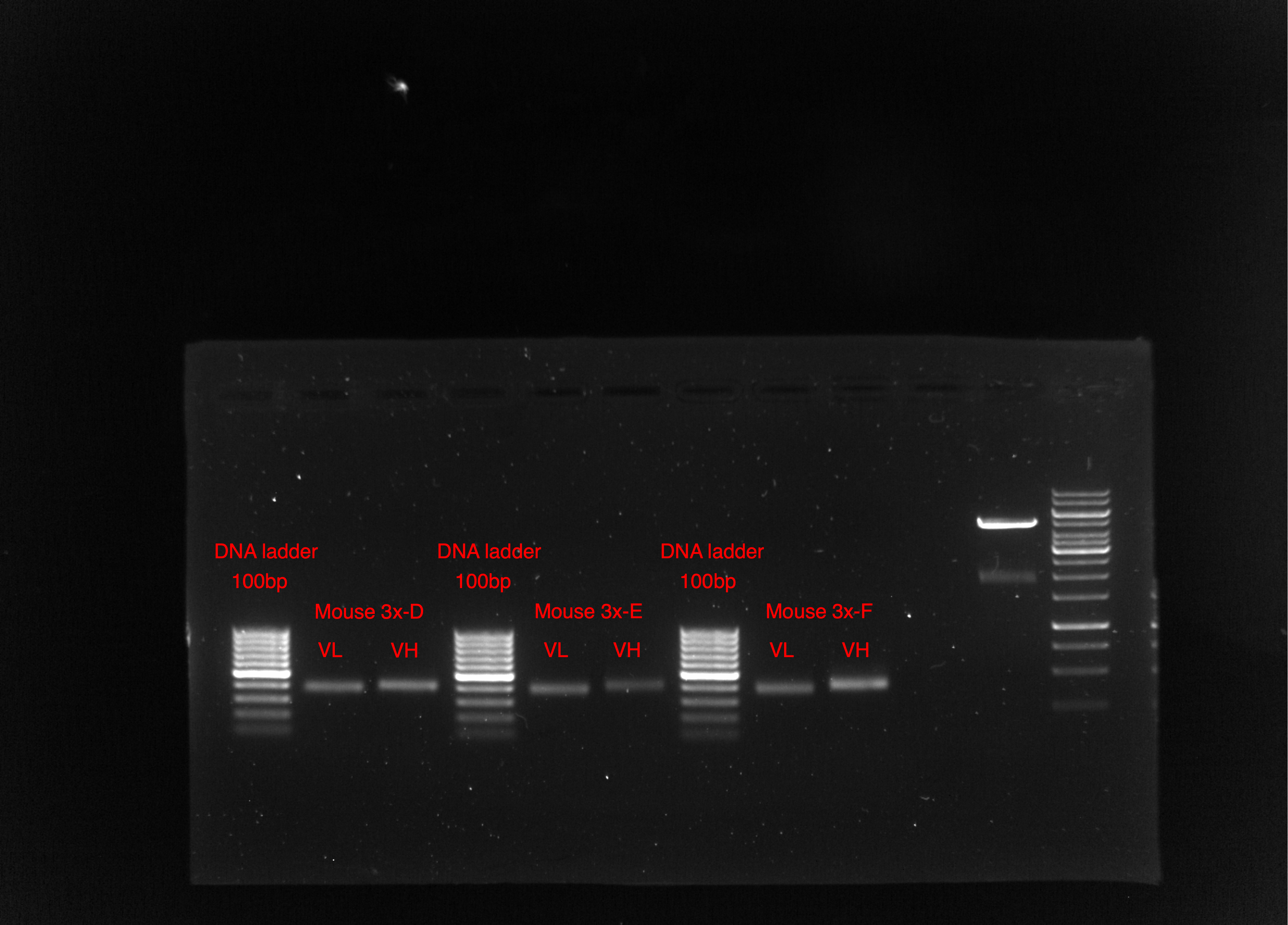

Supplement: Figure 5—figure supplement 1—source data 1. — Agarose gel electrophoresis (1%) of PCR-amplified VL and IgG VH genes from bone marrow (BM) samples of cohort-3x mice (3x-D, 3x-E, 3x-F), with 100 bp DNA size marker. [file elife-92718-fig5-figsupp1-data1.zip › Figure 5-figure supplement 1-source data 1/Figure 5-figure supplement 1-source data 1_labelled.tif]

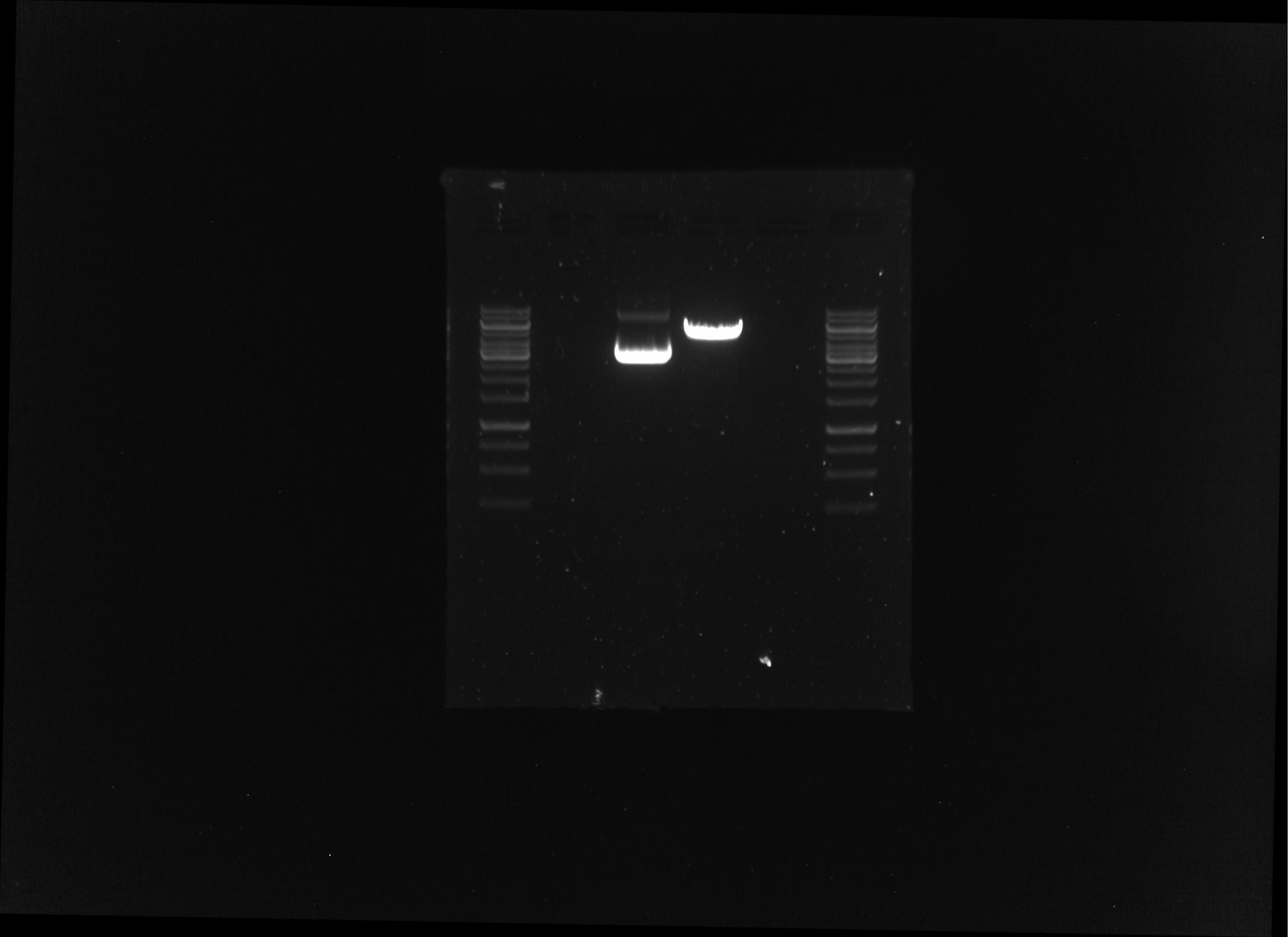

Supplement: Figure 5—figure supplement 1—source data 2. — Agarose gel electrophoresis (1%) of both unrestricted and BamHI-restricted pYD1 yeast surface display vectors, with 1 kb DNA size marker. [file elife-92718-fig5-figsupp1-data2.zip › Figure 5-figure supplement 1-source data 2/Figure 5-figure supplement 1-source data 2.tif]

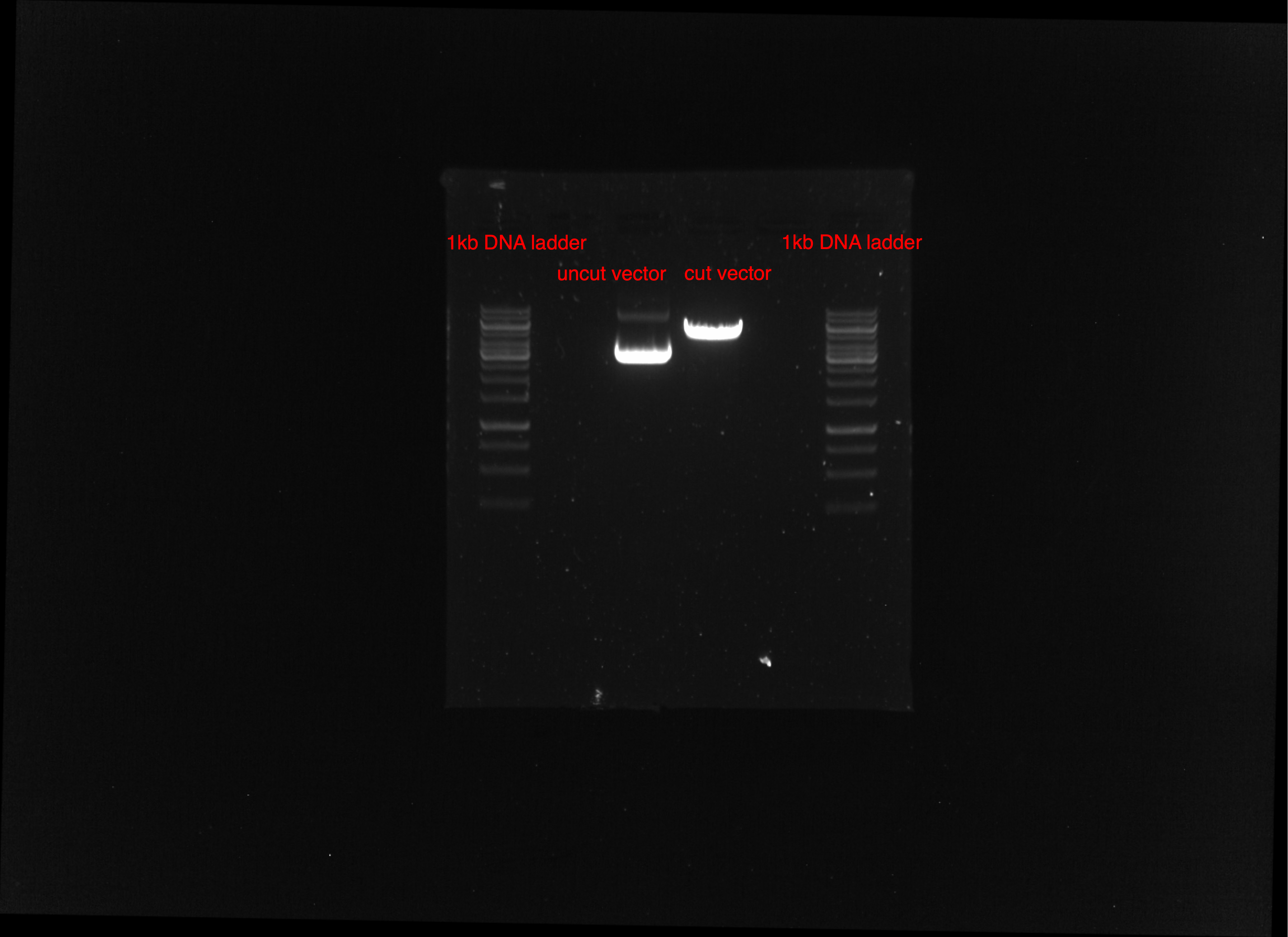

Supplement: Figure 5—figure supplement 1—source data 2. — Agarose gel electrophoresis (1%) of both unrestricted and BamHI-restricted pYD1 yeast surface display vectors, with 1 kb DNA size marker. [file elife-92718-fig5-figsupp1-data2.zip › Figure 5-figure supplement 1-source data 2/Figure 5-figure supplement 1-source data 2_labelled.tif]
