# Supplementary material for: The physiological landscape and specificity of antibody repertoires are consolidated by multiple immunizations
Source: eLife. 2024 Dec 18;13:e92718. doi: 10.7554/eLife.92718 (PMC11655063; doi:10.7554/eLife.92718)
Supplement: Supplementary file 1. — Numbers of quality-processed and length-trimmed sequencing reads as input into the MAF pipeline for error and bias correction for mice 1x-A, 1x-B, 1x-C, 3x-D, 3x-E, and 3x-F. BM: bone marrow; aLN-L, -R: left and right axillary lymph nodes; iLN-L, -R: left and right inguinal lymph nodes. [file elife-92718-supp1.docx]

Number sequence input into MAF pipeline

|  | **1x-A** | **1x-B** | **1x-C** | **3x-D** | **3x-E** | **3x-F** |
| --- | --- | --- | --- | --- | --- | --- |
| **aLN-L** | 405000 | 405000 | 405000 | 405000 | 405000 | 405000 |
| **iLN-L** | 405000 | 405000 | 405000 | 405000 | 405000 | 405000 |
| **aLN-R** | 281114 | 405000 | 405000 | 405000 | 405000 | 405000 |
| **iLN-R** | 16206 | 238220 | 405000 | 396147 | 405000 | 405000 |
| **spleen** | 405000 | 405000 | 405000 | 405000 | 405000 | 405000 |
| **BM** | 311438 | 336122 | 389748 | 405000 | 405000 | 405000 |
