# Supplementary material for: The physiological landscape and specificity of antibody repertoires are consolidated by multiple immunizations
Source: eLife. 2024 Dec 18;13:e92718. doi: 10.7554/eLife.92718 (PMC11655063; doi:10.7554/eLife.92718)
Supplement: Supplementary file 2. — Numbers of unique clones (and clonotypes; defined by antibody sequences possessing identical germline V- and J-genes and 90% CDRH3 a.a. identity and identical length) obtained after MAF processing. BM: bone marrow; aLN-L, -R: left and right axillary lymph nodes; iLN-L, -R: left and right inguinal lymph nodes. [file elife-92718-supp2.docx]

Unique clones after MAF processing

|  | **1x-A** | **1x-B** | **1x-C** | **3x-D** | **3x-E** | **3x-F** |
| --- | --- | --- | --- | --- | --- | --- |
| **aLN-L** | 740 (531) | 1055 (798) | 777 (629) | 630 (377) | 861 (407) | 760 (420) |
| **iLN-L** | 702 (480) | 615 (421) | 684 (513) | 419 (256) | 702 (348) | 794 (409) |
| **aLN-R** | 282 (250) | 323 (207) | 570 (461) | 457 (303) | 675 (361) | 481 (300) |
| **iLN-R** | 55 (50) | 263 (205) | 374 (289) | 419 (257) | 810 (435) | 389 (284) |
| **spleen** | 3025 (2573) | 3166 (2714) | 2916 (2370) | 2503 (1746) | 2475 (1618) | 2480 (1673) |
| **BM** | 1252 (1042) | 1264 (1080) | 1479 (1189) | 1520 (1049) | 1846 (1189) | 1665 (1099) |
