# Supplementary material for: The physiological landscape and specificity of antibody repertoires are consolidated by multiple immunizations
Source: eLife. 2024 Dec 18;13:e92718. doi: 10.7554/eLife.92718 (PMC11655063; doi:10.7554/eLife.92718)
Supplement: Supplementary file 3. — Table indicates overlap of the five most diverse clonotypes (top 1–top 5) across lymphoid organs within each mouse. The numbers represent organs (1: aLN-L; 2: iLN-L; 3: iLN-R; 4: aLN-R; 5: spleen; 6: BM) that share the same clonotypes, being among the top five in all indicated organs. BM: bone marrow; aLN-L, -R: left and right axillary lymph nodes; iLN-L, -R: left and right inguinal lymph nodes. [file elife-92718-supp3.docx]

|  |  | **Five most diverse clonotypes per organ** | | | | | | | | | |  |
| --- | --- | --- | --- | --- | --- | --- | --- | --- | --- | --- | --- | --- |
|  |  | **top1** | **top2** | | **top3** | | **top4** | | **top5** | |  |  |
| **Mouse 1x-A** | **aLN-L (1)** | 2 | |  | |  | |  | |  | | |
|  | **iLN-L (2)** |  | | 2 | |  | |  | |  | | |
|  | **iLN-R (3)** | 4 | | 4,5,6 | |  | |  | |  | | |
|  | **aLN-R (4)** | 3 | | 3,5,6 | |  | |  | |  | | |
|  | **spleen (5)** | 3,4,6 | | 6 | |  | |  | |  | | |
|  | **BM (6)** | 3,4,5 | |  | |  | | 5 | |  | | |
|  |  |  | |  | |  | |  | |  | | |
| **Mouse 1x-B** | **aLN-L (1)** |  | |  | |  | | 2 | | 2 | | |
|  | **iLN-L (2)** | 1 | |  | |  | | 1 | |  | | |
|  | **iLN-R (3)** | 4,6 | |  | | 4,5,6 | | 5,6 | |  | | |
|  | **aLN-R (4)** |  | |  | | 3,5,6 | |  | | 3,6 | | |
|  | **spleen (5)** | 6 | |  | | 3,4,6 | | 3,6 | |  | | |
|  | **BM (6)** | 3,4,5 | | 3,5 | |  | | 3,4 | | 5 | | |
|  |  |  | |  | |  | |  | |  | | |
| **Mouse 1x-C** | **aLN-L (1)** | 2,5,6 | |  | |  | |  | |  | | |
|  | **iLN-L (2)** | 2,5,6 | |  | |  | |  | |  | | |
|  | **iLN-R (3)** |  | |  | |  | |  | | 4 | | |
|  | **aLN-R (4)** |  | |  | |  | | 3 | |  | | |
|  | **spleen (5)** | 6 | | 1,2,6 | | 6 | | 6 | |  | | |
|  | **BM (6)** | 5 | | 1,2,5 | | 5 | | 5 | |  | | |
|  |  |  | |  | |  | |  | |  | | |
| **Mouse 3x-D** | **aLN-L (1)** | all | | all | | all | |  | |  | | |
|  | **iLN-L (2)** | all | | all | | all | |  | |  | | |
|  | **iLN-R (3)** | all | | all | |  | | all | |  | | |
|  | **aLN-R (4)** | all | | all | |  | |  | | all | | |
|  | **spleen (5)** | all | | 6 | | all | | 6 | | all | | |
|  | **BM (6)** | all | | all | | 5 | | 5 | | all | | |
|  |  |  | |  | |  | |  | |  | | |
| **Mouse 3x-E** | **aLN-L (1)** | 2,5,6 | | all | | all | | 2,4,5,6 | |  | | |
|  | **iLN-L (2)** | all | | all | |  | | 14,5,6 | | 1,5,6 | | |
|  | **iLN-R (3)** | all | | all | |  | |  | |  | | |
|  | **aLN-R (4)** | all | |  | | all | | 1,2,5,6 | |  | | |
|  | **spleen (5)** | all | | all | | 1,2,6 | | 1,2,4,6 | |  | | |
|  | **BM (6)** | all | | all | | 1,2,5 | | 1,2,4,5 | |  | | |
|  |  |  | |  | |  | |  | |  | | |
| **Mouse 3x-F** | **aLN-L (1)** |  | |  | | all | | 2,5,6 | | 2 | | |
|  | **iLN-L (2)** | all | | 5,6 | | 1 | | 1,5,6 | | 3,6 | | |
|  | **iLN-R (3)** | 4 | | 2,6 | |  | | 4,6 | | all | | |
|  | **aLN-R (4)** |  | |  | | 3 | | all | | 3,6 | | |
|  | **spleen (5)** | 1,2,6 | |  | |  | | all | | 2,6 | | |
|  | **BM (6)** | 1,2,5 | | all | | 2,5 | | 3,4 | | 2,3 | | |
