# Supplementary material for: The physiological landscape and specificity of antibody repertoires are consolidated by multiple immunizations
Source: eLife. 2024 Dec 18;13:e92718. doi: 10.7554/eLife.92718 (PMC11655063; doi:10.7554/eLife.92718)
Supplement: Supplementary file 4. — Numbers of FACS-isolated B-cell subsets per organ, as well as the yield of all cells and IgG+ B cells after single-cell V(D)J sequencing of antibody repertoires. [file elife-92718-supp4.docx]

| **Organ** | **CD19+ IgG+** | **CD19+ IgG-** | **PB/PC** | **10X cells** | **10X IgG cells** |
| --- | --- | --- | --- | --- | --- |
| **aLN-L** | 942 | 532 |  | 935 | 443 |
| **iLN-L** | 12950 |  |  | 4279 | 3416 |
| **spleen** | 15133 |  | 4861 | 4384 | 1431 |
| **BM** | 5996 |  | 10236 | 4074 | 888 |
