# Supplementary material for: The physiological landscape and specificity of antibody repertoires are consolidated by multiple immunizations
Source: eLife. 2024 Dec 18;13:e92718. doi: 10.7554/eLife.92718 (PMC11655063; doi:10.7554/eLife.92718)
Supplement: Supplementary file 5. — CDRH3 and CDRL3 information of IgG+ B cells shared among at least three organs, including cell counts for each organ. Black check marks indicate antibodies tested for RSV-F-binding as scFv format in yeast cells. Green check marks indicate confirmed RSV-F-binding of single-cell clones, whereas gray symbols indicate presumptive binding/non-binding of clones belonging to the corresponding clonotype. BM: bone marrow; aLN-L: left axillary lymph node; iLN-L: left inguinal lymph node. [file elife-92718-supp5.docx]

| **#** | **CDRH3** | **CDRL3** | **Cell counts** | | | | **Tested** | **Binder** |
| --- | --- | --- | --- | --- | --- | --- | --- | --- |
|  |  |  | **aLN-L** | **iLN-L** | **spleen** | **BM** |  |  |
| 1 | CKGFFDYFDFW | CFQGSHVPLTF |  | 1 | 1 | 2 | 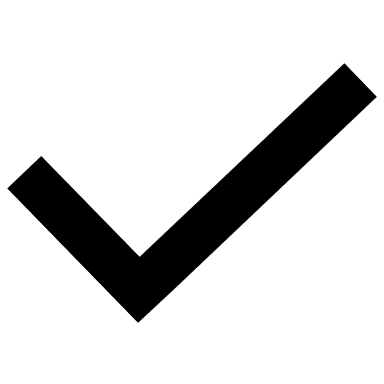 | 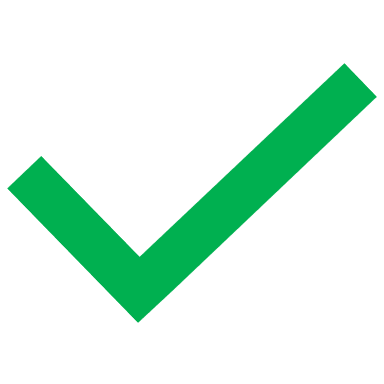 |
| 2 | CARTDSW | CWQGTHFPQTF |  | 1 | 3 | 2 |  | **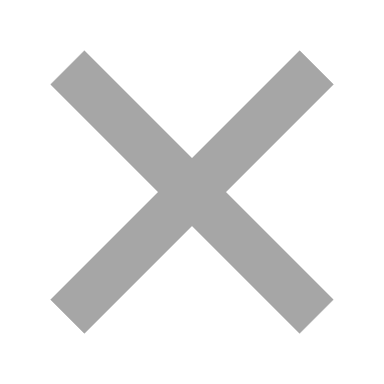** |
| 3 | CARTTYW | CWQGTHFPQTF |  | 1 | 2 | 6 | 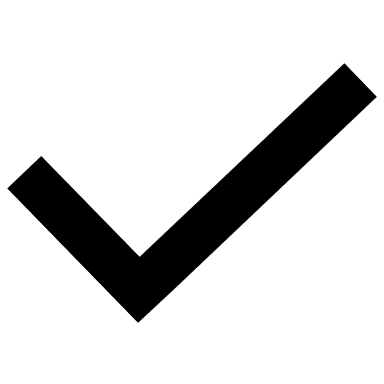 | **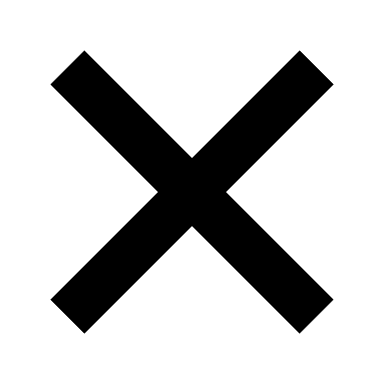** |
| 4 | CASPPLYDYDWFAYW | CSQSTHVPWTF |  | 7 | 1 | 8 | 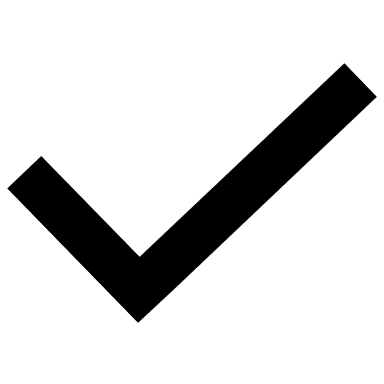 | **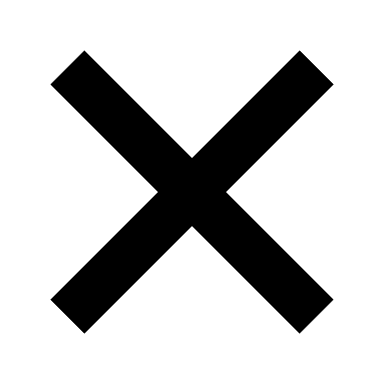** |
| 5 | CARVATAYWYFNVW | CQHHYGTPPTF |  | 3 | 1 | 5 | 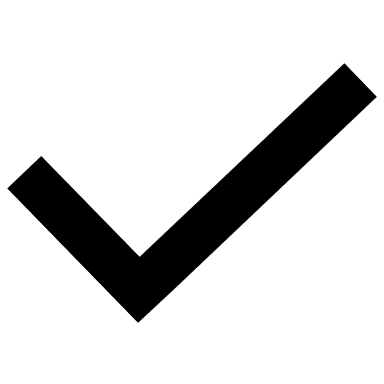 | **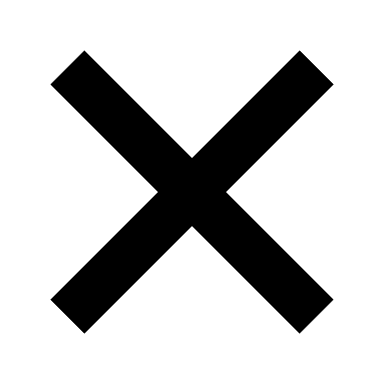** |
| 6 | CARLGLTRRYFDVW | CQQNNEDPWTF |  | 39 | 1 | 1 | 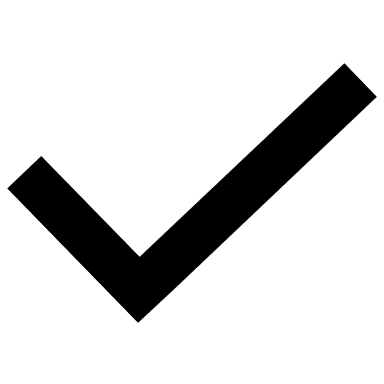 | **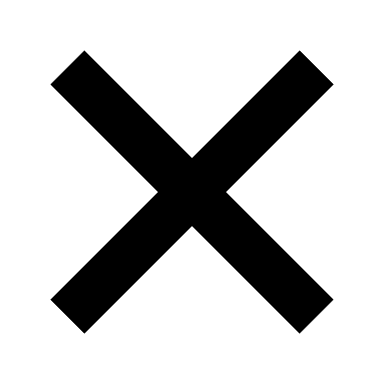** |
| 7 | CARKGYDVAWFAYW | CQQWSSNPLTF | 2 | 15 | 5 | 25 | 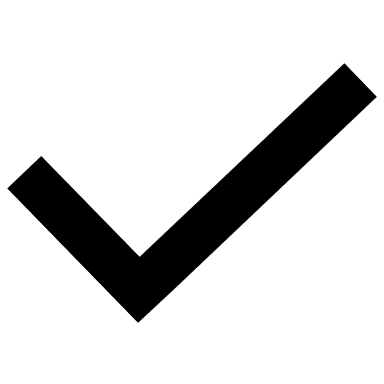 | **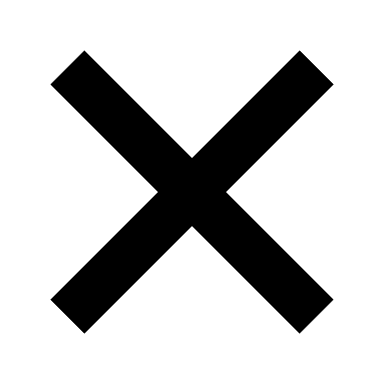** |
| 8 | CASIDYDYETFAYW | CQQRSSSPLTF |  | 1 | 3 | 8 | 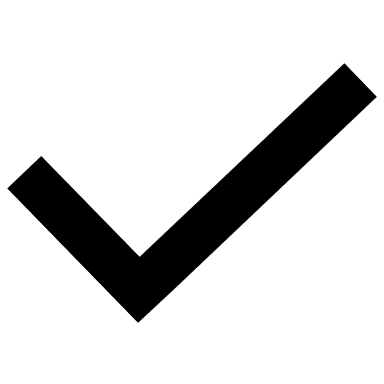 | **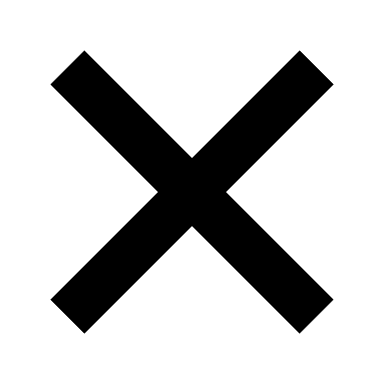** |
| 9 | CARSGGNYGAYYGMDYW | CQQWSSSPPTF |  | 39 | 3 | 1 |  | **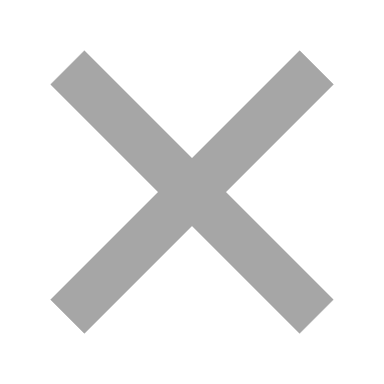** |
| 10 | CARSGGNYGAYYGMDYW | CQQWSSNPPTF | 2 | 2 | 1 | 4 | 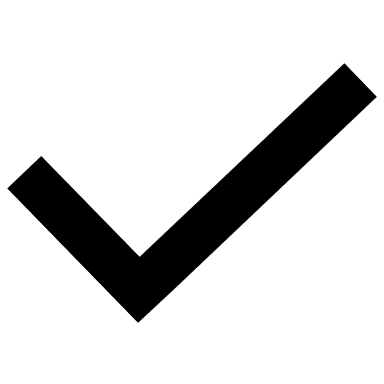 | **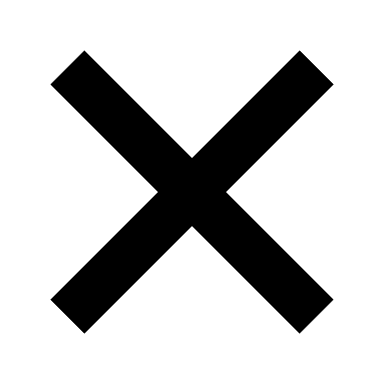** |
| 11 | CARHEEAGVKDHFDYW | CLQSDNMPFTF | 1 |  | 2 | 1 | 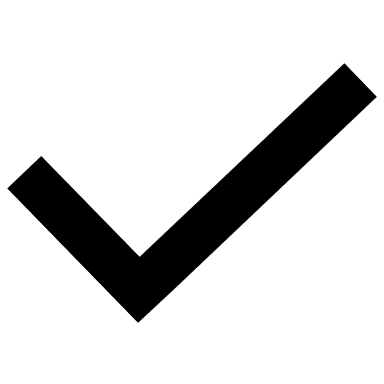 | **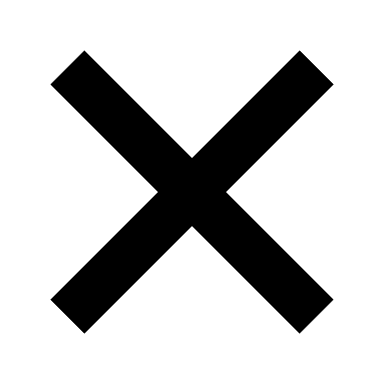** |
| 12 | CSRHETPAWFVYW | CQQTYNWPYTF |  | 7 | 4 | 3 |  | **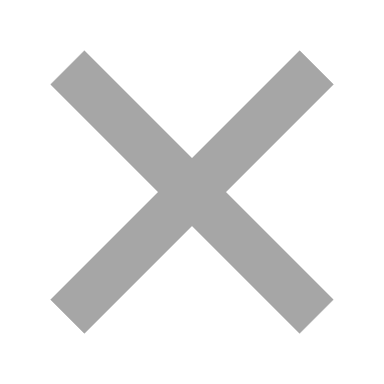** |
| 13 | CARHETPAWFAYW | CQQSNNWPYTF | 1 |  | 2 | 11 | 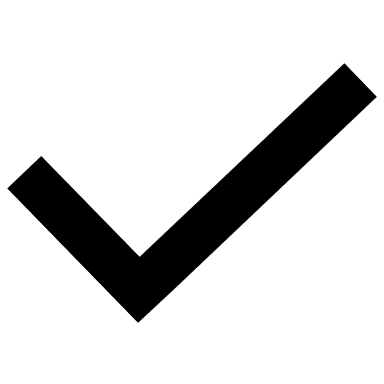 | **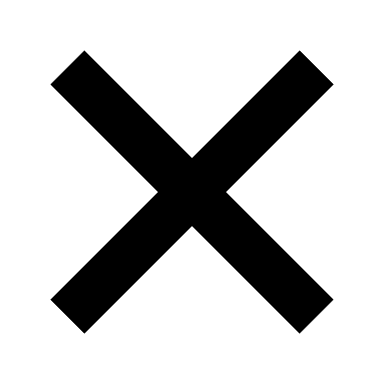** |
| 14 | CVSIAQPYYW | CALWYSNHFVF | 7 | 57 | 1 |  | 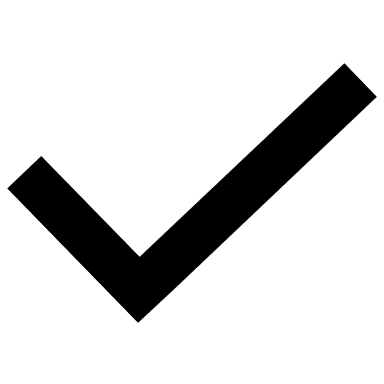 | 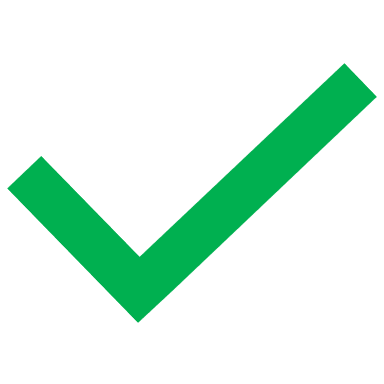 |
| 15 | CVRSDGNYFGYAMDYW | CHQYLFSYTF | 7 |  | 1 | 1 | 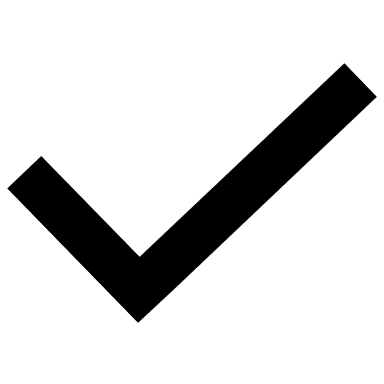 | **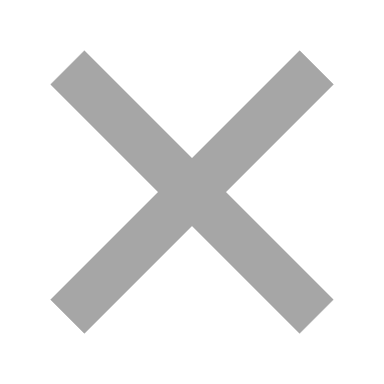** |
| 16 | CAREVYPYYFDYW | CAQNLELPWTF | 2 | 2 | 1 |  |  | 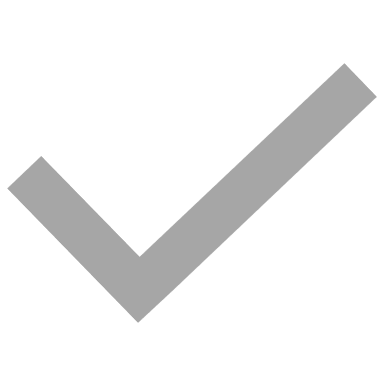 |
| 17 | CARELYPYYFDYW | CAQNLELPWTF | 10 | 277 |  | 14 | 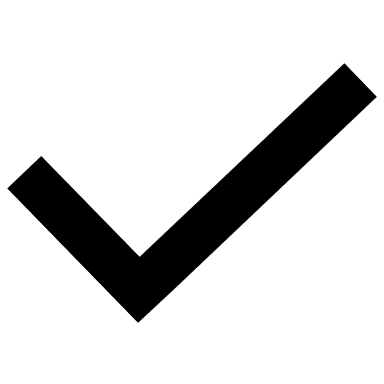 | 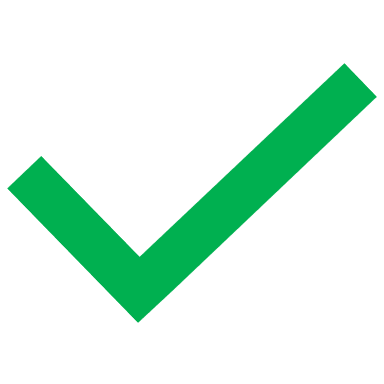 |
| 18 | CTRELYPYYFDYW | CAQNLELPWTF | 2 | 14 |  | 1 | 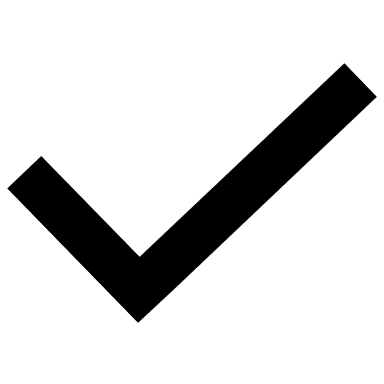 | 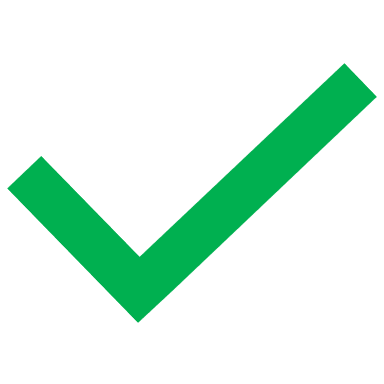 |
| 19 | CTRIYYGSPGFAYW | CAQNLELPWTF | 2 | 1 |  | 2 |  | 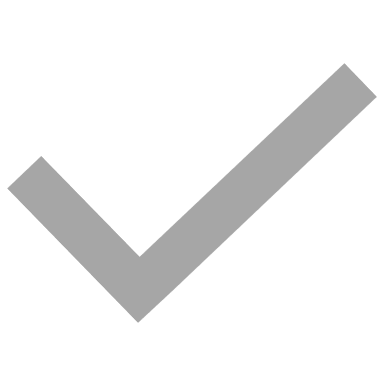 |
| 20 | CVRIYYGSPGFAYW | CAQNLELPWTF | 2 |  | 1 | 2 | 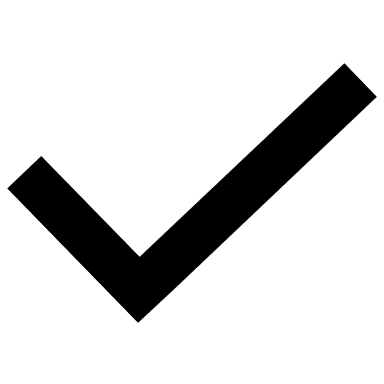 | 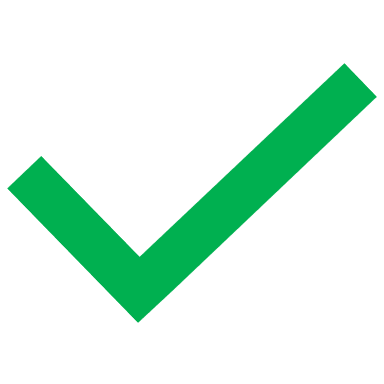 |
| 21 | CASELAWFAYW | CLQHVESPLTF | 2 |  | 1 | 8 | 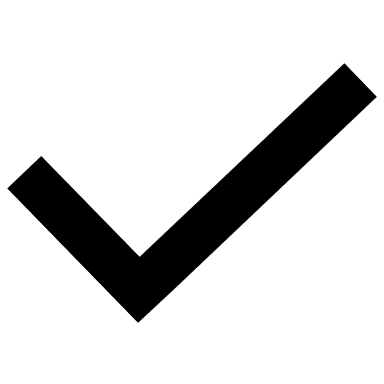 | **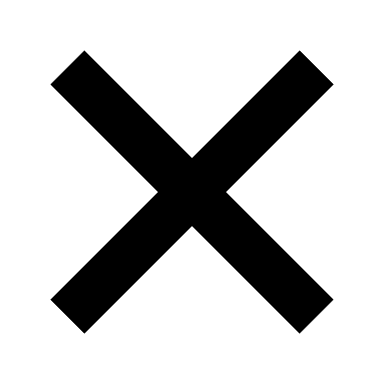** |
| 22 | CARRSYGSSMDYW | CQQGNSFPWTF |  | 15 | 1 | 2 |  | 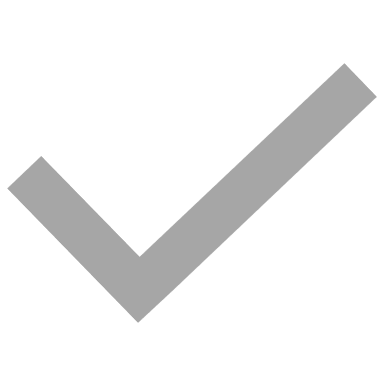 |
| 23 | CARRSYGSSMDYW | CQQGSTFPWTF |  | 11 | 1 | 4 |  | 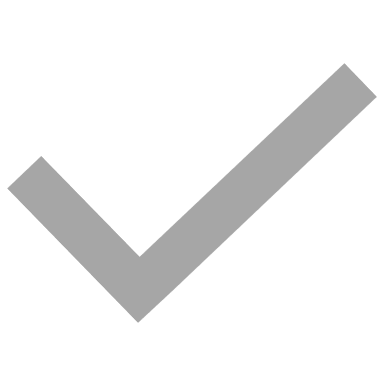 |
| 24 | CARRSYGSSMDYW | CQQGNTFPWTF |  | 16 | 1 | 18 | 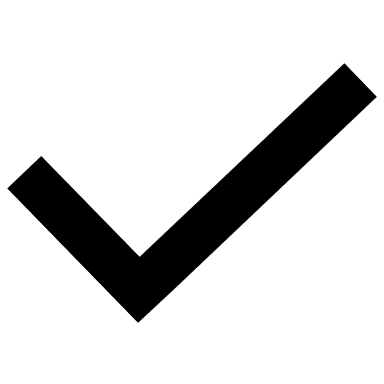 | 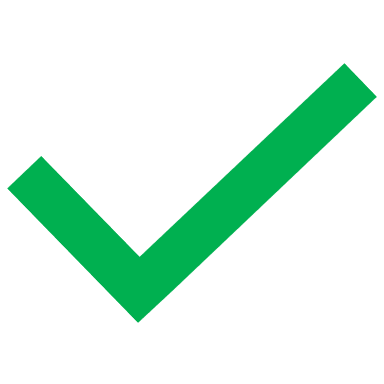 |
| 25 | CARRYYGSSMDSW | CQQGNTFPWTF |  | 1 | 2 | 3 |  | 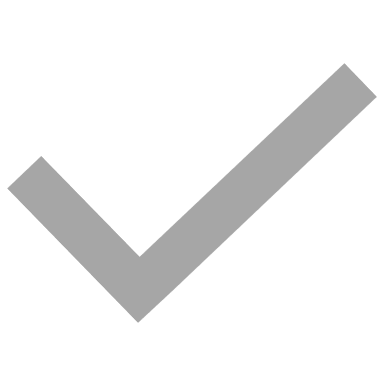 |
| 26 | CARYYRYRYFDYW | CQQGNTLPWTF |  | 90 | 2 | 7 | 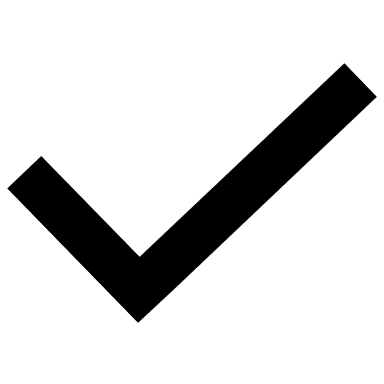 | **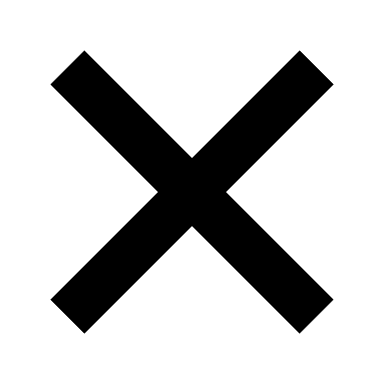** |
