# Supplementary material for: The physiological landscape and specificity of antibody repertoires are consolidated by multiple immunizations
Source: eLife. 2024 Dec 18;13:e92718. doi: 10.7554/eLife.92718 (PMC11655063; doi:10.7554/eLife.92718)
Supplement: Supplementary file 6. — VL-binding primers p3-26 were adapted from Reddy et al., 2010 and IgG- and VH-binding primers p1, 2, 27–45 were adapted from Khan et al., 2016. [file elife-92718-supp6.docx]

| Name | Binding region | Sequence (5’ to 3’) | Other |
| --- | --- | --- | --- |
| p1 (fwd) | HC | CCCTTGACCAGGCA | reverse transcription, IgG1, 2a, 2b |
| p2 (fwd) | HC | CCTTTGACAAGGCATCC | reverse transcription, IgG3 |
| p3 (fwd) | VL | TTCCAGACTACGCAGGATCC**GAYATCCAGCTGACTCAGCC** | multiplex PCR, overhang **binding** |
| p4 (fwd) | VL | TTCCAGACTACGCAGGATCC**GAYATTGTTCTCWCCCAGTC** | multiplex PCR, overhang **binding** |
| p5 (fwd) | VL | TTCCAGACTACGCAGGATCC**GAYATTGTGMTMACTCAGTC** | multiplex PCR, overhang **binding** |
| p6 (fwd) | VL | TTCCAGACTACGCAGGATCC**GAYATTGTGYTRACACAGTC** | multiplex PCR, overhang **binding** |
| p7 (fwd) | VL | TTCCAGACTACGCAGGATCC**GAYATTGTRATGACMCAGTC** | multiplex PCR, overhang **binding** |
| p8 (fwd) | VL | TTCCAGACTACGCAGGATCC**GAYATTMAGATRAMCCAGTC** | multiplex PCR, overhang **binding** |
| p9 (fwd) | VL | TTCCAGACTACGCAGGATCC**GAYATTCAGATGAYDCAGTC** | multiplex PCR, overhang **binding** |
| p10 (fwd) | VL | TTCCAGACTACGCAGGATCC**GAYATYCAGATGACACAGAC** | multiplex PCR, overhang **binding** |
| p11 (fwd) | VL | TTCCAGACTACGCAGGATCC**GAYATTGTTCTCAWCCAGTC** | multiplex PCR, overhang **binding** |
| p12 (fwd) | VL | TTCCAGACTACGCAGGATCC**GAYATTGWGCTSACCCAATC** | multiplex PCR, overhang **binding** |
| p13 (fwd) | VL | TTCCAGACTACGCAGGATCC**GAYATTSTRATGACCCARTC** | multiplex PCR, overhang **binding** |
| p14 (fwd) | VL | TTCCAGACTACGCAGGATCC**GAYRTTKTGATGACCCARAC** | multiplex PCR, overhang **binding** |
| p15 (fwd) | VL | TTCCAGACTACGCAGGATCC**GAYATTGTGATGCBCAGKC** | multiplex PCR, overhang **binding** |
| p16 (fwd) | VL | TTCCAGACTACGCAGGATCC**GAYATTGTGATAACYCAGGA** | multiplex PCR, overhang **binding** |
| p17 (fwd) | VL | TTCCAGACTACGCAGGATCC**GAYATTGTGATGACCCAGWT** | multiplex PCR, overhang **binding** |
| p18 (fwd) | VL | TTCCAGACTACGCAGGATCC**GAYATTGTGATGACACAACC** | multiplex PCR, overhang **binding** |
| p19 (fwd) | VL | TTCCAGACTACGCAGGATCC**GAYATTTTGCTGACTCAGTC** | multiplex PCR, overhang **binding** |
| p20 (fwd) | VL | TTCCAGACTACGCAGGATCC**GARGCTGTTGTGACTCAGGAATC** | multiplex PCR, overhang **binding** |
| p21 (rev) | VL | CTGATCCACCGCCTCCACTCCCGCCACCTCC**TTTGATTTCCAGCTTGG** | multiplex PCR, (G_4_S)_3_ linker **binding** |
| p22 (rev) | VL | CTGATCCACCGCCTCCACTCCCGCCACCTCC**TTTTATTTCCAGCTTGG** | multiplex PCR, (G_4_S)_3_ linker **binding** |
| p23 (rev) | VL | CTGATCCACCGCCTCCACTCCCGCCACCTCC**TTTTATTTCCAACTTTG** | multiplex PCR, (G_4_S)_3_ linker **binding** |
| p24 (rev) | VL | CTGATCCACCGCCTCCACTCCCGCCACCTCC**TTTCAGCTCCAGCTTGG** | multiplex PCR, (G_4_S)_3_ linker **binding** |
| p25 (rev) | VL | CTGATCCACCGCCTCCACTCCCGCCACCTCC**TAGGACAGTCAGTTTGG** | multiplex PCR, (G_4_S)_3_ linker **binding** |
| p26 (rev) | VL | CTGATCCACCGCCTCCACTCCCGCCACCTCC**TAGGACAGTGACCTTGG** | multiplex PCR, (G_4_S)_3_ linker **binding** |
| p27 (fwd) | VH | GTGGAGGCGGTGGATCAGGTGGAGGAGGCTCT**GAGGTGAAGCTTCTCGAGTC** | multiplex PCR, (G_4_S)_3_ linker **binding** |
| p28 (fwd) | VH | GTGGAGGCGGTGGATCAGGTGGAGGAGGCTCT**GAGGTGCAGCTTGTTGAGTC** | multiplex PCR, (G_4_S)_3_ linker **binding** |
| p29 (fwd) | VH | GTGGAGGCGGTGGATCAGGTGGAGGAGGCTCT**CAGATCCAGTTGGTGCAGTC** | multiplex PCR, (G_4_S)_3_ linker **binding** |
| p30 (fwd) | VH | GTGGAGGCGGTGGATCAGGTGGAGGAGGCTCT**GAAGTGCAGCTGTTGGAGAC** | multiplex PCR, (G_4_S)_3_ linker **binding** |
| p31 (fwd) | VH | GTGGAGGCGGTGGATCAGGTGGAGGAGGCTCT**CAGGT/ ideoxyI/CAGCTGCAGCAGYC** | multiplex PCR, (G_4_S)_3_ linker **binding** |
| p32 (fwd) | VH | GTGGAGGCGGTGGATCAGGTGGAGGAGGCTCT**CAGGTTM/ ideoxyI/GCTGCAACAGTC** | multiplex PCR, (G_4_S)_3_ linker **binding** |
| p33 (fwd) | VH | GTGGAGGCGGTGGATCAGGTGGAGGAGGCTCT**CAGGTYCA/ideoxyI/CT/ideoxyI/CAGCAGTC** | multiplex PCR, (G_4_S)_3_ linker **binding** |
| p34 (fwd) | VH | GTGGAGGCGGTGGATCAGGTGGAGGAGGCTCT**CAGGTGCAGCTGAAGSAGTC** | multiplex PCR, (G_4_S)_3_ linker **binding** |
| p35 (fwd) | VH | GTGGAGGCGGTGGATCAGGTGGAGGAGGCTCT**GAGGTGCAGCTTCAGGAGTC** | multiplex PCR, (G_4_S)_3_ linker **binding** |
| p36 (fwd) | VH | GTGGAGGCGGTGGATCAGGTGGAGGAGGCTCT**GAAGTGAA/ideoxyI/CTTGAGGWGTC** | multiplex PCR, (G_4_S)_3_ linker **binding** |
| p37 (fwd) | VH | GTGGAGGCGGTGGATCAGGTGGAGGAGGCTCT**CAGGTTACTCTGAAAGAGT** | multiplex PCR, (G_4_S)_3_ linker **binding** |
| p38 (fwd) | VH | GTGGAGGCGGTGGATCAGGTGGAGGAGGCTCT**CAGAT/ideoxyI/CAGCTT/ideoxyI/AGGAGTC** | multiplex PCR, (G_4_S)_3_ linker **binding** |
| p39 (fwd) | VH | GTGGAGGCGGTGGATCAGGTGGAGGAGGCTCT**GAGGTG/ideoxyI/AGCTGGTGGAGTC** | multiplex PCR, (G_4_S)_3_ linker **binding** |
| p40 (fwd) | VH | GTGGAGGCGGTGGATCAGGTGGAGGAGGCTCT**GAGGTGCAGCTTGTAGAGAC** | multiplex PCR, (G_4_S)_3_ linker **binding** |
| p41 (fwd) | VH | GTGGAGGCGGTGGATCAGGTGGAGGAGGCTC**TCAGGT/ideoxyI/CAGCTGCAGCAGCC** | multiplex PCR, (G_4_S)_3_ linker **binding** |
| p42 (rev) | VH | TCATCTTTATAATCGGATCC**TGAGGAAACGGTGACCGTGGT** | multiplex PCR, overhang **binding** |
| p43 (rev) | VH | TCATCTTTATAATCGGATCC**TGAGGAGACTGTGAGAGTGGT** | multiplex PCR, overhang **binding** |
| p44 (rev) | VH | TCATCTTTATAATCGGATCC**TGCAGAGACAGTGACCAGAGT** | multiplex PCR, overhang **binding** |
| p45 (rev) | VH | TCATCTTTATAATCGGATCC**TGAGGAGACGGTGACTGAGGT** | multiplex PCR, overhang **binding** |
| p46 (fwd) | Pali-VL | TTCCAGACTACGCAGGATCC**GACATCCAGATGACCCAGAGC** | singleplex PCR, overhang **binding** |
| p47 (rev) | Pali-VL | CTGATCCACCGCCTCCACTCCCGCCACCTCC**CTTGATCTCCAGCTTGGTGCC** | singleplex PCR, (G_4_S)_3_ linker **binding** |
| p48 (fwd) | Pali-VH | GTGGAGGCGGTGGATCAGGTGGAGGAGGCTCT**CAGGTGACCCTGAGGGAGAGCG** | singleplex PCR, (G_4_S)_3_ linker **binding** |
| p49 (rev) | Pali-VH | TCATCTTTATAATCGGATCC**GCTGCTCACGGTCACGGTGG** | singleplex PCR, overhang **binding** |
| p50 (fwd) | (G_4_S)_3_ linker | TCGTCGGCAGCGTCAGATGTGTATAAGAGACAG**ATCAGGTGGAGGAGGCTC** | NGS PCR, partial adaptor **binding** |
| p51 (rev) | FLAG-tag | GTCTCGTGGGCTCGGAGATGTGTATAAGAGACAG**ATCGTCGTCATCTTTATAATCGG** | NGS PCR, partial adaptor **binding** |
